# Quality of Care Indicators for Head and Neck Cancers: The Experience of the European Project RARECAREnet

**DOI:** 10.3389/fonc.2019.00837

**Published:** 2019-08-28

**Authors:** Annalisa Trama, Laura Botta, Roberto Foschi, Otto Visser, Josep Maria Borras, Tina Žagar, Maja Primic-Žakelj, Francesca Bella, Nadya Dimitrova, Gemma Gatta, Lisa Licitra

**Affiliations:** ^1^Evaluative Epidemiology Unit, Research Department, Fondazione IRCCS Istituto Nazionale dei Tumori, Milan, Italy; ^2^Netherlands Comprehensive Cancer Organisation (IKNL), Utrecht, Netherlands; ^3^Department of Clinical Sciences, The Bellvitge Biomedical Research Institute (IDIBELL), University of Barcelona, Barcelona, Spain; ^4^Epidemiology and Cancer Registry, Institute of Oncology Ljubljana, Ljubljana, Slovenia; ^5^Integrated Cancer Registry of Catania-Messina-Siracusa-Enna, Azienda Ospedaliero-Universitaria Policlinico-Vittorio Emanuele, Catania, Italy; ^6^National Hospital of Oncology, Bulgarian National Cancer Registry, Sofia, Bulgaria; ^7^Department of Oncology and Hemato-Oncology, University of Milan, Milan, Italy; ^8^Head and Neck Cancer Medical Oncology Unit, Department of Medical Oncology, Fondazione IRCCS Istituto Nazionale dei Tumori, Milan, Italy

**Keywords:** head and neck cancers, population based studies, quality of care, quality indicators, integrated care

## Abstract

**Background:** Monitoring and improving quality of cancer care has become pivotal today. This is especially relevant for head and neck cancers since the disease is complex, it needs multi therapy, patients tend to be older, they tend to have comorbidities and limited social support. However, information on quality of care for head and neck cancers is scarce. In the context of the project “Information Network on Rare Cancers” we aimed to identify indicators of quality of care specific for the head and neck cancers management and to measure the quality of care for head and neck cancers in different EU Member States.

**Methods:** We defined indicators of quality of care for head and neck cancers based on a multidisciplinary and expert-based consensus process at a European level. To test the proposed indicators, we performed an observational population-based retrospective study in four countries (Ireland, Italy, Netherlands, and Slovenia) in the years 2009–2011.

**Results:** The main quality indicators identified are: availability of formalized multidisciplinary team, participation in clinical and translational research; timeliness of care, high quality of surgery and radiotherapy, and of pathological reporting. For head and neck cancers, the quality of care did not reach the optimal standards in most of the countries analyzed. A high proportion of patients was diagnosed at an advanced disease stage, showed delays in starting treatment (especially for radiotherapy), and there was only a very limited use of multi therapy.

**Conclusions:** According to the achieved consensus, indicators of quality of care for head and neck cancers have to cover the patient journey (i.e., diagnosis and treatment). Our results, showed suboptimal quality of care across countries and call for solutions for ensuring good quality of care for head and neck cancer patients in all EU countries. One possible option might be to refer head and neck cancer patients to specialized centers or to networks including specialized centers.

## Introduction

Due to the complexity of oncological care, it is essential to deliver integrated care with optimal alignment and collaboration of several disciplines (1). This is especially relevant for head and neck cancers (HNCs). HNCs involve different anatomical sites, including larynx, oral cavity, oropharynx, hypopharynx, nasopharynx, nasal cavity, and sinuses. The disease is complex, often needs multi therapy, including surgery, radiation, chemotherapy, and/or targeted therapy. Patients tend to be older, to have comorbidities and less social support (2).

In order to improve high-quality oncological care, reliable quality indicators (QIs) are indispensable. This was first highlighted in the USA by the Institute of Medicine's report, “Crossing the Quality Chasm: A New Health System for the twenty-first Century” (3). In Europe, reviews on QI of cancer care were made available by the European Partnership for Action Against Cancer (http://www.epaac.eu) and by the European CanCer Organisation Essential Requirements for Quality Cancer Care (ERQCC) (https://www.ecco-org.eu/Global/News/Latest-News/2017/02/ECCO-Essential-Requirements-for-Quality-Cancer-Care). With regards to HNCs, quality assurance (QA) has been extensively addressed for HNC radiotherapy (RT) (4, 5) and surgery (6, 7) and to a lesser extent for medical oncology (1, 8–11).

Against this background, in the framework of the Information network on rare cancers (RARECAREnet) project (www.rarecarenet.eu), we defined QIs to measure quality of oncological care for the HNC patient journey (i.e., diagnosis and treatment).

In this paper, we report on: (1) the QIs of cancer care for HNC identified by RARECAREnet; (2) the results of the study testing the proposed QIs in several EU countries.

## Materials and Methods

Quality indicators for HNC were defined through a consensus process following the steps below:

1. Identification of published QIs;
2. Discussion of the proposed QIs with an expert panel involving multidisciplinary experts in a dedicated meeting;
3. Population-based observational study in several European countries to test the proposed QIs;
4. Discussions about the results of the observational assessment with the same expert panel in a second meeting in which a final agreement on QIs was achieved with no dissent. During this second meeting, the expert panel confirmed the QIs originally proposed and added new indicators. For the latter, data were not collected because the observational assessment had ended. We refer to them as QIs agreed by consensus only (Table 1).

**Table 1:** List of quality indicators for head and neck cancers.

**Diagnostic management**
1. Percentage of patients with a defined stage at diagnosis
**Time to start treatment** (12) **and treatment adherence to clinical guidelines** (13)
2. Time to start treatment (time between definitive pathological diagnosis and beginning of surgery or radiotherapy <1 month) 3. Time in starting post-operative radiotherapy or concomitant chemo-radiotherapy (<8 weeks from surgery)
4. Percentage of patients with early stage I and II referred for either surgery or radiotherapy 5. Percentage of patients with locally advanced stage III and IV referred for surgery plus post-operative radiotherapy or post-operative chemo-radiotherapy or concomitant chemo-radiotherapy
**Quality of surgery and radiotherapy**
6. Percentage of complete tumor resection (histological verification of tumor free margins after surgery) 7. Percentage of re-operation within 30 days from main surgery^*^ 8. Percentage of grade ≥3 late toxicities (>3 months after radiotherapy)^*^ 9. Percentage of patients receiving intensity-modulated radiation therapy vs. % receiving 3D conformal radiation therapy 10. Percentage of patients receiving the appropriate surgery for its stage (e.g., minimal invasive, reconstructive surgery)^*^
**Quality of pathology reports after surgery**
11. Percentage of pathology reports after surgery with a full set of core data items recorded. According to the Royal College of Pathologists (https://www.rcpath.org/profession/publications/cancer-datasets.html): site and laterality of the carcinoma, maximum diameter of tumor, maximum depth of invasion, histological type of carcinoma, degree of differentiation (grade), pattern of invasion, margin status, lymph node involvement.
Availability of formalized multidisciplinary decision (with member experts on head and neck cancers)^*^
Participation in clinical and translational research^*^

* *Indicators agreed by consensus within the expert panel only*.

The HNC multidisciplinary expert panel included faculty members of the European Society for Medical Oncology (ESMO), authors of the ESMO clinical practice guidelines (CPG), representatives of the European Head and Neck Society (EHNS), of the European Society for Radiotherapy and Oncology (ESTRO) and representatives of the European Society of Surgical Oncology (ESSO). HNC patients were represented by the Italian association of laryngectomized patients (Associazione Italiana Laringectomizzati), which is a member of the Make Sense Campaign of the EHNS, and by the European Cancer Patient Coalition.

The observational assessment was performed in collaboration with population-based cancer registries (CRs): the national CRs of Ireland, Netherlands, and Slovenia, as well as nine Italian regional CRs. The study included only incident squamous cell carcinoma (SCC) of larynx, oral cavity, oropharynx, and hypopharynx, diagnosed in patients >15 years old. In Ireland and Netherlands all incident SCC of larynx, oral cavity, oropharynx, and hypopharynx were included in the study. In Slovenia, the study focused only on larynx however, all incident cases of larynx SCC diagnosed in Slovenia were included. In Italy, due to the lack of a national CR, nine CRs representative of the different incidence rates of HNCs considered were included. These nine CRs included all incident cases of larynx, oral cavity, oropharynx, and hypopharynx SCC diagnosed in the geographical areas covered by the selected nine CRs. Table 2 enlists the number of cases included by country and the years of diagnosis.

**Table 2:** Number of patients with head and neck cancers (HNCs) included in the study by country with years of diagnosis.

**Cancer**	**Number**				
	**Total**	**Ireland**	Italy^*^	**Netherlands**	**Slovenia**
HNCs	8,655	1,323	928	6,185	219
Hypopharynx	790	121	54	615	0
Larynx	3,168	449	398	2,102	219
Oral cavity	2,976	428	258	2,290	0
Oropharynx	1,722	325	218	1,178	0
Years of diagnosis		2009–2011	2009–2010	2009–2011	2009–2010

* *Italy included nine population based cancer registries: Registro Tumori Integrato (Catania and Messina), Palermo, Ragusa, and Siracusa (Sicily-south of Italy); Modena, Parma, Reggio Emilia, and Romagna (Emilia Romagna-centre of Italy); Friuli Venezia Giulia (Friuli Venezia Giulia-north est of Italy)*.

We developed a data collection protocol with HNC clinical experts and CRs, we established a help desk to answer questions on the data collection and we centralized at the Istituto Nazionale Tumori (INT) data quality checks and analyses. All data obtained from CRs were fully anonymized prior to being accessed centrally at INT. The Ethics Committee of INT was notified about the RARECAREnet project including this retrospective observational study.

For HNCs, clinical stage was adapted from the ESMO CPG (13) breaking down patient populations into:

- Localized: T1-T2, N0, M0
- Advanced: T3-T4, N0, M0, or N+ with any T
- Metastatic: M+
- Unknown.

The treatment combinations for HNCs were defined as follows:

- Surgery alone; RT alone; chemotherapy alone (if each started within 3 months from diagnosis),
- Concomitant chemo-radiotherapy (CRT) (if chemotherapy and radiotherapy started on the same day, or if chemotherapy started 1 day before the radiotherapy or within max 21 days after the start of radiotherapy),
- Surgery + radiotherapy or surgery + concomitant radio-chemotherapy (concomitant if chemotherapy and radiotherapy started on the same day, or chemotherapy started 1 day before the radiotherapy or within max 21 days after the start of radiotherapy),
- Other combinations of treatments,
- No treatment.

## Results

Table 1 summarizes the RARECAREnet quality of care indicators for HNCs. All QIs are listed i.e., QI agreed by consensus and assessed by the observational study and QIs agreed by consensus only. It was agreed that optimum management of HNC patients requires active involvement of experts from a wide variety of fields including at least: a head and neck surgeon, a radiation oncologist, a pathologist, a radiologist and a medical oncologist, high quality of surgery and radiotherapy, timely start of treatment (12), optimal supportive care management, and the ability to manage complex patients with multiple health and social care needs.

The observation study was performed on 1,323, 928 and 6,185 cases of hypopharynx, larynx, oral cavity, and oropharynx SCC in Ireland, Italy, and Netherlands, respectively. In Slovenia only larynx SCC were included in study (*N* = 219) (Table 2).

Tables 3–5 summarize the results of the observational assessment for the QIs included in the study by country.

**Table 3:** Diagnostic management for head and neck cancers illustrated for larynx and other sites of the head and neck (i.e., hypopharynx, oral cavity and oropharynx), by country.

**Diagnostic management**										
**Country**	**Indicator 1. Percentage of patients with a defined stage at diagnosis**									
	**Larynx**					**Other sites**				
	***N***	**% L**	**% A**	**% M+**	**% Missing**	***N***	**% L**	**% A**	**% M+**	**% Missing**
Ireland	449	40	35	3	22	874	17	55	4	24
Italy	398	50	30	4	16	530	24	49	8	19
Netherlands	2,102	58	37	1	4	4,083	38	55	2	4
Slovenia	219	54	41	1	4					

*Indicator 1: Number (N) of cases overall and percentage (%) of patients diagnosed with localized (L), advanced (A), and metastatic (M+) disease together with % of cases with information on stage missing, by country*.

Regarding staging at diagnosis (Table 3), in Netherlands and Slovenia, almost all patients were staged (unknown stage <5%). In Italy and Ireland, stage was unknown in about one out of six and one out of four patients, respectively. Most of hypopharynx, oropharynx, and oral cavity cancer patients were diagnosed with an advanced disease stage across all countries.

Table 4 reports the results for the timeliness in starting treatment and the adherence to CPG (Indicators 2, 3, 4, and 5). Many HNC patients started treatment with curative intent (surgery or RT) >1 month after the diagnosis. In Italy, 60% of HNC patients started the treatment within 1 month from diagnosis, in all the other countries the proportion starting the treatment on time decreased to 40%. Most of the HNC patients started adjuvant treatment <8 weeks after surgery ranging from 52% in Italy to 79% in Netherlands. The only exception was Ireland with 33% of HNC patients starting adjuvant within the recommended number of days. Adherence to CPG was high for HNC patients with localized disease stage (Indicator 4: 72–79%) but low for HNC patients with advanced disease stage (Indicator 5: 19–44%). Differences were observed in the use of surgery and radiotherapy across head and neck sites and countries. In Italy, surgery was the main treatment for all HNCs, in the other countries RT was the main treatment for larynx cancers and surgery for the other sites (data available from the corresponding author).

**Table 4:** Time to start treatment and treatment adherence to clinical practice guidelines by country.

**Time to start treatment and treatment adherence to clinical practice guidelines**										
**Country**	**Indicator 2. Time to start RT or surgery**		**Indicator 3. Time in starting adjuvant therapy**		**Indicator 4% of patients with L disease stage treated with surgery or RT**			**Indicator 5% of patients with A disease stage treated with multi therapy**		
	***N*** **treated with surgery or RT**	**% starting surgery or RT** **<1 month from diagnosis**	***N*** **with adjuvant therapy**	**% starting adjuvant therapy** **<8 weeks from the surgery**	**N with L disease**	**% treated with surgery alone or RT alone**	**M%**	**N with A disease**	**% treated with multi therapy**	**M%**
Ireland	1,055	41	202	33	332	72	8	640	43	5
Italy	595	61	94	52	327	75	13	380	19	15
Netherlands	4,741	39	900	79	2,792	79	10	3,005	21	22
Slovenia	198	41	69	57	119	72	3	90	44	3

Indicator 2: Number (N) of head and neck cancer patients treated with surgery or radiotherapy (RT), percentage (%) of patients treated with surgery or RT starting surgery or RT <1 month from diagnosis. Indicator 3: Number (N) of head and neck cancer patients treated with adjuvant therapy^*^ percentage (%) of head and neck patients starting adjuvant therapy <8 weeks from the surgery. Indicator 4: Number (N) of head and neck cancer patients with localized disease (L), percentage (%) of head and neck cancer patients with localized (L) disease treated with surgery alone or RT alone and percentage (%) of missing (M) information for this indicator. Indicator 5: Number (N) of head and neck cancer patients with advanced disease (A), percentage (%) of head and neck cancer patients with advanced (A) disease treated with multi therapy^**^ and percentage (%) of missing (M) information for this indicator. ^*^Adjuvant therapy: post-operative radiotherapy or post-operative concomitant chemo-radiotherapy. ^**^Multi therapy: surgery plus post-operative radiotherapy or post-operative chemo-radiotherapy or concomitant chemo-radiotherapy alone.

Table 5 describes the quality of surgery and of the pathological report after surgery (Indicators 6 and 11). CRs did not find adequate information on type of RT thus results are not reported for the indicator 9. The proportion of HNC patients with complete tumor resection after a surgery with curative intent, ranged from 56% in Ireland to >70% in Netherland and Slovenia. The resection margin was unknown in 27% of cases in Ireland and in <15% of cases in the other countries. The pathological report after surgery included all necessary information in a minority of cases (from 1% in Slovenia to 24% in Italy). However, in most cases (80–90%), at least site and laterality of the tumor, histological type and grade were reported. The information less often described were maximum depth of invasion and the pattern of invasion (data available from the corresponding author).

**Table 5:** Quality of surgery and of the pathological report after surgery.

**Quality of surgery and of the pathological report after surgery**						
**Country**	**Indicator 6% of complete tumor resection**			**Indicator 11% of pathology reports with all core data**		
	***N*** **treated with surgery with curative intent**	**% of R0**	**M%**	***N*** **surgically treated**	**% of post-surgery pathological report with all core data**	**M%**
Ireland	602	56	27	NA	NA	
Italy	516	62	13	474	24	2
Netherlands	2,728	74	13	128^**^	16^**^	0
Slovenia	88	75	9	87	1	29

Indicator 6: Number (N) of head and neck patients treated with surgery with curative intent^*^, percentage (%) of head and neck patients treated with surgery with negative resection margins (R0) and percentage (%) of missing (M) information for this indicator. Indicator 11: Number (N) of head and neck patients treated with surgery (excluding cryotherapy photodynamic therapy or electrocautery procedure, cryosurgery or laser therapy or thermo-ablation), percentage (%) of pathological report after surgery with all the information available and percentage (%) of missing (M) information for this indicator. ^*^Surgery with curative intent: surgery alone or surgery + adjuvant treatment.

*NA, not available*.

** *Evaluated on a sample of 200 randomly selected case*.

## Discussion

We proposed QIs of cancer care for HNCs based on a multidisciplinary and expert-based consensus process at European level. The proposed QIs cover two critical steps of the patient journey (i.e., diagnosis and treatment) and are easy to collect at the hospital as well as at the population level (i.e., from CRs or administrative data sources). Previous QA for HNCs focused on surgery (6, 7) and RT (4, 5). In addition, extensive research have supported the important role of multidisciplinary team (MDT) care for HNCs (2, 14–17).

The indicators proposed for surgery include: the presence of a multidisciplinary tumor board advising on more than 90% of HNCs, the capacity to perform all necessary imaging, the existence of clinical pathways, collaboration with paramedical services, institutional guidelines' hygiene standards being monitored by an institutional board, clinical trial data managers, reports on surgical procedures as indicated by the American Academy of Otolaryngology, Head and Neck Surgery, pathology reports as indicated by the Royal College of Pathologists dataset for histopathology reporting, established reporting system for undesirable events (7).

General radiation oncology (RO)-QIs measure efficiency, waiting time, accuracy of medical records, percentage of cases discussed in a MDT setting, treatment planning based on CT, frequency of verification of treatment portal, measures of physics quality control adequacy, and patient satisfaction (18). For HNC, the National Quality Measures Clearinghouse identified two radiation oncology-related QI i.e., complete follow-up documented for patients receiving RT for glottic cancer and patients receiving post-operative head and neck RT 6 weeks after surgery or longer (4). Guidelines for the delineation of the primary tumor clinical target volumes are also available (19–21). In addition, for HNCs, it is internationally agreed that RT-QA is important, that a radiation oncologist should not practice HNC without adequate training, and that a RT-QA program should be available in RO departments treating HNC. To date, there is no strict international consensus exists on the best model of RT-QA, neither in relation to HNC nor otherwise. However, the benefits of RT-QA and peer review suggest that this has to be incorporated into routine clinical practice. Technology is evolving at a rapid rate: machine learning, artificial intelligence, deformable registration, and radiomics may bring additional refinements to the peer review process. Peer review will form an important component of adaptive treatment, but before this is implemented we will need to consider how to best add this additional burden to head and neck departments (5).

Our QIs include the quality of both, surgery and RT and support the importance of MDT care, of timely start of treatment and of the quality of the pathology reports. A limitation of our QIs is that they do not address quality in systemic therapy. Quality assurance in the medical arena arrived most recently in comparison to other fields. At the beginning of the 90s, the European organization for research and treatment of cancer (EORTC) addressed issues related to the practice of chemotherapy delivery and the quality of data reporting. Furthermore, the EORTC quality assurance committee proposed a minimal set of quality control procedures to be implemented by all EORTC groups (10). However, QA for medical oncology in HNCs was not developed further. A major problem in HNCs medical oncology is the dose intensity in multi therapy. HNC patients with locally advanced disease stage treated with CRT experience moderate/severe side-effects limiting their tolerance to receive the intended cisplatin dose intensity (22). Chemotherapy modifications (dose reductions/delays/omissions) are common (23–40%) (23, 24). The consequence of cumulative cisplatin dose reduction is uncertain although some reports suggest a possible detrimental impact on survival (22, 23, 25). Discrepancies in treatment adherence expressed as proportion of patients who received all planned cycles of chemotherapy could therefore be used as a QI for systemic therapy in HNC. In this regard, there is an important role for the clinician, not only to stimulate patients to adhere to the treatment schedule, but also to provide optimal supportive care in order to make it tolerable for the patient (22).

Previous studies looking at the quality of integrated care for HNC patients proposed structure and process indicators (e.g., availability of MDT, of an integrated care pathway, of a case manager, of electronic patient information system) (26, 27). Other studies proposed an extensive list of outcome, process and structure indicators that needed to be practically tested so to limit the QIs number (1). Although interesting, these studies report about one country-specific context. We tested the QIs in an observational study performed in four different European countries. The data collection, based on clinical dossiers and administrative database, was undertaken with an acceptable proportion of missing (<20–25%) in all countries and without major problems in the data collection but for 1 RT indicator. The expert panel agreed that the results of the observational study gave a good description of the quality of care for HNCs in the studies of all the countries, confirming the reliability and validity of the QI proposed in measuring quality of care.

We found that quality of care of HNC does not reach optimal standards in some of the countries analyzed. Many patients were diagnosed an advanced disease stage, which is associated with a worse prognosis (28). Another major problem was treatment delay. This happened most likely when patients were treated with RT, which could impact on their prognosis (29). Possible explanations include possible delays in referral to RO and concentration of radiotherapy facilities in a few centers with limited resources. A recent survey on radiotherapy capacities in Europe showed significant variability among countries and a lack of RT infrastructures (30). The adherence to CPG for treatment was good. However, we observed a very limited use of multi therapy for advanced-stage patients. This is relevant considering that they are associated with a higher survival (31). We observed inadequate surgery and poor quality of the pathological reports after surgery, which is a matter of concern considering that pathological reports support treatment decisions after surgery.

Our study adds evidence to previous national studies in which compliance of HNCs care to hospital or national CPG was considered a quality care marker. A Dutch study reported a compliance rate of 91% (32), one study from the USA 86% (33), going down to 57% in patients with persistent or recurrent HNCs who were referred to an expert centre (34).

Limitations of our study include the retrospective design and the potential errors in coding associated with the kind of sources of clinical data and their inherent variability in quality of reporting, although every effort was made to standardize data collection and reduce missing data. Strengths are the centralization of data quality checks and analyses along with the population-based nature of this effort, which is essential to generate real-world data.

Our results showed suboptimal standards of quality of care in some of the countries analyzed and call for solutions to increase quality care for HNC patients to ensure high quality of care across EU countries. One way of increasing quality of care for HNC patients is to refer them to specialized centers or networks involving specialized centers. Thus, the available evidence show that in the high volume context quality of care is ensured in the entire patient journey:

1. The specialized MDT, which takes considerable time, effort and financial resources works better (35); furthermore the presence of a MDT in high-volume and referral cancer centers is associated with better therapeutic decision (35);
2. The minimal level of quality of surgery is most likely to be reached considering the structural and process criteria identified by the QA programme and the number of major head and neck procedures that should be performed by a leading surgeon per year (7),
3. Experienced clinicians are available to deliver complex HNC RT treatment most accurately and to peer review RT complex plans ensuring RT-QA program (5).

Furthermore, high volume seems to be associated also with better outcomes for HNCs (36–38).

Centralization for many rare cancers including HNCs is still limited in many countries in Europe (39). Gatta et al. reported that for HNCs, 75% of patients were centralized in two top hospitals in Slovenia (2 million population, 266 treatments per hospital per year), and 12 top hospitals in the Netherlands (17 million population, 201 treatments per hospital per year). The level of centralization was lower in the other countries included in the study, resulting in a caseload of 145 treatments/year on overage in each of the 10 Bulgarian top hospitals, 106 treatments/year on average in each of the 29 top Belgian hospitals, 83 treatments/year on average in each of the top six hospitals in Finland, 77 treatments/year on average in the two top hospitals in Navarra, and 63 treatments/year on average in the top seven hospitals in Ireland (39).

It follows that the centralization of care, although hardly feasible for all HNC patients, should be an objective to be pursued. We strongly believe that this objective can only be achieved by progressively making it a national health care policy priority.

The quality ensured by the case volume could be explained by several factors: organization, facilities, processes of care, quality assurance programs, professional expertise, adherence to clinical protocols, technology. In this context, it will be important to detect which of these factors influences final outcomes among HNC patients. We are currently performing additional analyses to assess the relationship between hospital volume and the QIs proposed. We are also trying to assess whether the proposed QIs can explain the observed higher survival observed in high volume centers. In this paper, we present the QI and the process behind their definition. Furthermore, we provide data on quality of care for HNC patients across four countries. This study may be a starting point showing the variability in clinical practice as well as the need to make every effort to increase quality of care for HNC patients in all EU countries.

## Data Availability

All datasets generated for this study are included in the manuscript and/or the supplementary files.

## Ethics Statement

The study was approved by the Ethics Committee of the IRCCS National Cancer Institute. Being a retrospective study on a rare and lethal disease, the written consent was not necessary.

## Author Contributions

AT: study design, supervision of data collection, results interpretation, and manuscript writing. LB and RF: study design and statistical analyses. OV: study design, data collection, results interpretation, and manuscript revision. JB: manuscript revision. TŽ, MP-Ž, and FB: data collection, results interpretation, and manuscript revision. ND, GG, and LL: study design, results interpretation, and manuscript revision.

### Conflict of Interest Statement

The authors declare that the research was conducted in the absence of any commercial or financial relationships that could be construed as a potential conflict of interest.

## The RARECAREnet High Resolution Working Group

**Belgian Cancer Registry:** Elizabeth Van Eycken, Kris Henau; **Bulgaria National Cancer Registry:** Nadia Dimitrova; **Finland National Cancer Registry:** Maarit Leinonen; **Ireland National Cancer Registry**: Harry Comber, Fiona F. Dwane; **Italy: Registro tumori del Friuli Venezia Giulia:** Silvia Birri, Diego Serraino; **Registro tumori della provincia di Modena:** Claudia Cirilli, Martina Manni; **Registro tumori della provincia di Parma**: Maria Michiara, Anita Rimanti; **Registro tumori Reggiano**: Lucia Mangone, Massimo Vicentini; **Registro tumori della Romagna:** Fabio Falcini, Stefania Giorgetti; **Registro tumori Integrato (RTI) di CT-ME-SR-EN:** Salvatore Sciacca, Francesca Bella; **Registro tumori di Palermo:** Rosanna Cusimano, Rosalba Amodio; **Registro tumori ASP Ragusa per le provincie di Ragusa e Caltanissetta:** Rosario Tumino, Patrizia Concetta Rollo; **Registro tumori della provincia di Siracusa:** Anselmo Madeddu, Antonino Ziino Colanino; **Registro tumori di Trapani:** Giuseppa Candela, Tiziana Scuderi; **Slovenia National Cancer Registry**: Maja Primic-Žakelj, Tina Žagar; **The Netherlands** Otto Visser, Jan Maarten van der Zwan; **Fondazione IRCCS Istituto Nazionale dei Tumori, Milano**: Nadia Hindi, Martina Imbimbo, Riccardo Capocaccia.
